# Patient perspectives on delays in cervical cancer screening and follow-up care in Botswana: a mixed methods study

**DOI:** 10.1186/s12905-022-01777-z

**Published:** 2022-05-28

**Authors:** Katharine A. Rendle, Doreen Ramogola-Masire, Barati Monare, Shannon N. Ogden, Hannah K. Toneff, Chelsea A. Saia, Jocelyn V. Wainwright, Tara M. Friebel-Klingner, Lisa Bazzett-Matabele, Rohini Bhatia, Natalie Bonner, Tlotlo B. Ralefala, Peter Vuylsteke, Rebecca Luckett, Surbhi Grover

**Affiliations:** 1 Department of Family Medicine and Community Health, University of Pennsylvania, Philadelphia, PA USA; 2 Department of Biostatistics, Epidemiology, and Informatics, University of Pennsylvania, Philadelphia, PA USA; 3 Department of Obstetrics and Gynecology and Office of Research and Graduate Studies, University of Botswana, Gaborone, Botswana; 4 Botswana-University of Pennsylvania Partnership, Gaborone, Botswana; 5 Department of Health Law, Policy, and Management, Boston University, Boston, MA USA; 6 Department of Epidemiology, Johns Hopkins Bloomberg School of Public Health, Baltimore, MD USA; 7 Department of Obstetrics and Gynecology, University of Botswana, Gaborone, Botswana; 8 Department of Radiation Oncology and Molecular Sciences, Johns Hopkins School of Medicine, Baltimore, MD USA; 9 UT Southwestern Medical School, Dallas, TX USA; 10 Department of Oncology, Princess Marina Hospital, Gaborone, Botswana; 11 Department of Internal Medicine, University of Botswana, Gaborone, Botswana; 12 Botswana-Harvard AIDS Institute Partnership, Gaborone, Botswana; 13 Department of Obstetrics and Gynecology, Beth Israel Deaconess Medical Center, Boston, MA USA; 14 Department of Radiation Oncology, University of Pennsylvania, Philadelphia, PA USA

**Keywords:** Global health, Cervical cancer, Early detection, HIV, Mixed methods study

## Abstract

**Background:**

Delays in screening and timely diagnosis contribute significantly to global disparities in cervical cancer mortality in Botswana and other low- and middle-income countries, particularly those with high rates of HIV. Little is known about the modifiable factors shaping these delays from the perspectives of women themselves and how these perspectives may differ between those living with and without HIV.

**Methods:**

From March–May 2019, we conducted a concurrent, mixed methods study of women receiving treatment for cervical cancer at a multidisciplinary oncology clinic in Botswana. Enrolled participants completed a one-time, concurrent semi-structured interview and structured questionnaire assessing patient characteristics, screening and HIV-related beliefs and knowledge, and barriers and facilitators to screening and follow-up care. Qualitative data were analyzed using directed content analysis guided by the Model of Pathways to Treatment and triangulated with quantitative questionnaire data to identify areas of convergence and divergence. Fisher’s exact tests were used to explore associations between questionnaire data (e.g., screening knowledge) and HIV status.

**Results:**

Forty-two women enrolled in the study, 64% of whom were living with HIV and 26% were diagnosed with stage III cervical cancer. Median age was 45 years (IQR 54–67) in those living with HIV and 64 years (IQR 42–53) in those living without. Overall screening rates before symptomatic disease were low (24%). Median time from most proximal screen to diagnosis was 52 median days (IQR 15–176), with no significant differences by HIV status. General screening knowledge was higher among those living with HIV versus those without (100% vs 73%; *p* < 0.05), but knowledge about HPV and other risk factors was low in both groups. Similar to questionnaire results, qualitative results indicate limited awareness of the need to be screened prior to symptoms as a central barrier to timely screening. Some participants also noted that delays in the receipt of screening results and fear also contributed to treatment delays. However, many participants also described myriad sources of social and tangible support that helped them to overcome some of these challenges.

**Conclusion:**

Interventions focused on increasing routine screening and supporting timely awareness and access to care are needed to reduce global disparities in cervical cancer.

## Background

Cervical cancer is the fourth leading cause of cancer mortality in females globally with over 600,000 new cases and 300,000 deaths estimated in 2020 alone [1]. The vast majority (approximately 90%) of cervical cancer cases and deaths occur in low- and middle-income countries (LMICs), particularly those with high rates of HIV as cervical cancer is an AIDS-defining malignancy [1]. Botswana is burdened with high rates of cervical cancer incidence (34.4 per 100,000) and mortality (20.1 per 100,000) and HIV (18.5%) [1]. Despite a robust HIV care infrastructure and a national program in cervical cancer control that provides free screening at community and clinic sites throughout the country [2–4], the majority of patients with cervical cancer in Botswana present with locally advanced disease, driven partly by suboptimal implementation and delays in evidence-based care across the cancer control continuum [5, 6]. This is particularly concerning given that cervical cancer is considered to be nearly completely preventable and often curable if treated early [6, 7].

In high-income countries, cervical cancer screening and timely access to treatment has dramatically reduced incidence and mortality from this preventable and curable disease [2, 3]. While screening coverage in high income countries is around 63%, screening coverage in LMICs averages 19% [4]. Previous research in LMICs have identified a number of challenges to successful implementation of cervical cancer screening programs including limited resources for pathology with resulting delays in receipt of results and limited access to treatment, both of which contribute to advanced disease at diagnosis and disease progression prior to treatment [5, 6]. Despite significant progress and investment in HIV treatment and cervical cancer screening programs in Botswana both by the Government of Botswana and donors, incidence of cervical cancer remains very high [7, 8]. Most cervical cancer patients present at advanced stages and about half of the women with cervical cancer report that they have never been screened for cervical cancer [8–10]. While previously published literature points to lack of cervical cancer awareness among women in Botswana, little is known about how individual, community, or system-level factors shape delays across screening and diagnostic pathways from the perspective of women themselves or how delays may be amplified or attenuated for women living with HIV [11, 12].

Our study was conducted to gain deeper understanding into the persistence of high rates of advanced stage of cervical cancer at presentation despite increased efforts towards cervical cancer screening and diagnostic follow-up in Botswana. Here we describe and compare patient-reported facilitators and barriers to timely screening of cervical cancer among women living with and without HIV who were diagnosed with cervical cancer in Botswana.

## Methods

The goal of this mixed methods study was to understand patient-reported factors shaping delays in screening and follow-up among patients presenting for cervical cancer care at a multidisciplinary oncology clinic in Botswana. Specifically, we employed a concurrent mixed methods design, in which enrolled participants simultaneously completed both a structured questionnaire and semi-structured interview at the time of enrollment. This method was used to triangulate and compare cross-cutting domains of inquiry collected qualitatively and quantitatively [13]. Participants interviewed in this study were part of a larger clinical cohort study of over 1,000 patients with cervical cancer designed to assess longitudinal treatment outcomes [10, 14].

### Setting

Recruitment and interviews took place at a public multidisciplinary oncology clinic in Botswana that provides free cancer care for patients. This clinic is the only one of its kind in the country and sees an estimated 85% of all patients with cervical cancer in Botswana [9]. Most patients in this clinic present with locally advanced disease warranting radiation. Despite an estimated population of 2 million, Botswana has a single radiation oncology facility with one linear accelerator and one brachytherapy unit, located near the recruiting clinic. Prior to starting active treatment, patients with histologically confirmed cervical cancer are referred to this oncology clinic and team for overall care management.

### Eligibility and recruitment

From March–May 2019, we approached potentially eligible patients presenting for cervical cancer care at the oncology clinic. Patients were eligible for study enrollment if they had been diagnosed with cervical cancer (any stage), attended at least one clinical visit at the recruiting clinic, and agreed to participate. In this care setting, new patients often do not have scheduled appointments but are rather seen based on when they arrive; thus, women often come early and wait in the designated waiting room. Depending on the clinic load, women may be seen immediately or wait for several hours. As is customary to this setting, the clinic usually begins with a short hymn and prayer followed by discussion of any cancer-related topics, led by the clinical research manager (MM). During this group discussion, the research coordinator introduced the study to all patients and then reviewed the charts for eligibility for any women who expressed interest in being interviewed. Eligible patients were given an informational consent statement about the study and asked if they were interested in participating. Patients who agreed to participate completed study activities (interview plus structured questionnaire) that day in a private room in the clinic. All study activities were approved by the institutional review boards at University of Pennsylvania, Princess Marina Hospital, and Ministry of Health and Wellness of the Republic of Botswana prior to study commencement.

### Data collection

After receiving written informed consent, a research team member (clinical study coordinator) who has experience conducting interviews in Setswana and English completed data collection activities in the participants’ preferred language. We used a concurrent mixed methods design to collect data which included simultaneous completion of a structured quantitative questionnaire and an audio-recorded semi-structured qualitative interview [13]. The structured questionnaire was available in separate written form, but all participants selected to have the questionnaire administered orally by the interviewer. All participants were asked the same survey and interview questions. Following approximately five interviews, an expert in mixed methods research (KR), reviewed the transcripts of the qualitative portion of the study and provided feedback to help refine the interview process and content. Interviews lasted approximately 30 min on average and were professionally transcribed and back-translated to English for analysis. Survey data were entered directly into a secure survey platform (REDCap) for data management [15, 16].

### Quantitative measures

The structured questionnaire captured participant sociodemographic information (e.g., education, marital status, number of children, cell phone ownership, place of residence), and assessed beliefs and knowledge related to cervical cancer risk, screening, and HIV. Drawing upon similar studies conducted in sub-Saharan Africa, we used existing items to measure general cervical cancer screening awareness, human papillomavirus (HPV) and cervical cancer prevention and screening knowledge, and experiences of HIV and cervical cancer screening stigma [17]. General cervical cancer screening awareness (e.g., “Have you ever heard of cervical cancer?” and “Have you ever heard of HPV or human papillomavirus?”) were measured using seven items with either a “Yes,” “No,” or “No Response.” HPV and cervical cancer screening knowledge was measured by 15 statements to which the participants responded as “True,” “False,” or “I don’t know.” Some example statements included, “Women should get screened for cervical cancer only if they have symptoms,” “Having HIV increases a woman's risk of cervical cancer,” and “HPV is an infection that can cause cervical cancer.” Experiences of HIV and cervical cancer screening stigma (e.g., “Do you feel you were rejected by family?” and “Did you become a social outcast?”) were captured separately by the same eight items with responses of “Yes,” “No,” or “No Response” [17]. Additional data were abstracted from medical records regarding clinical factors, such as stage, pathology, screening history, and treatment dates. For the assessment of general HIV awareness, and HIV transmission and prevention knowledge, we used items from a prior survey conducted among adults in Botswana [18]. All of these items had “Yes,” “No,” or “I don’t know” response options. Table 2 includes verbatim language of survey items included in analysis.

### Qualitative interview guide

Guided by the domains across the Model of Pathways to Treatment, a widely used framework developed by Scott and colleagues to assess phases of treatment seeking and completion [19], the semi-structured qualitative interview consisted of open-ended questions designed to gather additional insights on participants’ understanding of cervical cancer screening, HPV, and HIV and to capture experiences with cervical cancer screening and follow-up care. The interview guide consisted of questions related to (1) knowledge of cervical cancer, cervical cancer screening, HPV, and HIV; (2) decision-making factors in regard to cervical cancer screening and treatment receipt; (3) cervical cancer diagnosis and treatment experiences; and (4) health information and care seeking behaviors [8]. The semi-structured guide was systematically used to interview all participants, allowing for consistent interrogation of domains across and within participants. In this manuscript, we focus on the experiences up to cancer diagnosis. We ended data collection after reaching data saturation, which was established after hearing recurrent themes across participants and comparing our results to prior research on delays in care [20].

### Mixed methods data analysis

For quantitative analysis, we first used descriptive statistics to summarize survey data collected from all participants. We used clinical data on prior screening dates, biopsy dates, and treatment dates to identify delays in care. To assess bivariate associations between patient characteristics, beliefs, and knowledge, and HIV status, we used Fisher’s exact test for categorical variables and Student t-tests for continuous variables. All statistical analyses were conducted using STATA 15, and *p* < 0.05 were considered statistically significant.

For the qualitative analysis, we used directed content analysis and grounded theory to deductively and inductively identify themes across and within participants [21]. First, we developed and applied a codebook guided by the Model of Pathways to Treatment to capture patient, community, clinical and system-level factors contributing to the delays to cervical cancer screening during each phase of the decisional and behavioral processes prior to beginning treatment (appraisal, help-seeking, diagnostic phases) [19]. Then, we utilized modified grounded theory to identify additional concepts in the data, such as perspectives on health and healthcare generally [22]. Throughout the coding stages, the team developed and refined a codebook to apply specific codes to the data that aligned with the various decisional and behavioral factors related to cervical cancer screening and diagnostic care. The codebook finalization and analysis underwent an iterative process where the research team conferred and agreed upon the code definitions and resulting themes. Upon finalization, a doctoral-level team member (SO) applied the full codebook to all transcripts using qualitative software (NVivo Version 12, QSR International). Using a concurrent approach, we triangulated qualitative themes and quantitative survey data to identify areas of convergence and divergence [21].

### Patient and public involvement

Local clinical stakeholders in Botswana were involved in the development, execution, and evaluation of this study. Outside of enrolled participants, there was no direct patient involvement in this study.

## Results

### Patient characteristics

A total of 42 women undergoing cervical cancer treatment enrolled in the study, of which 64% were living with HIV at the time of diagnosis (Table 1). Median age was significantly lower (*p* < 0.001) in women living with HIV (45 years) compared to those without HIV (64 years). Women with HIV were more likely to present with earlier stage of disease compared to women without HIV, with 47% of women living with HIV presenting with Stage III in comparison to 15% of women living without HIV (*p* = 0.01). Most women interviewed had a primary education level or lower (40%), identified as Christian (90%), and preferred Setswana as their primary language (83%). In comparison to women living without HIV, women living with HIV were significantly more likely to use public transportation (96% vs 80%; *p* < 0.05) and more likely to be single (85% vs 53%, *p* < 0.05) at the time of the interview.

**Table 1:** Participant sociodemographic and clinical characteristics by HIV status

Characteristics (n, column %)	Total	Characteristics by HIV status		
	All participants (N = 42)	Living without HIV (N = 15)	Living with HIV (N = 27)	*P* value
Living with HIV	27 (64%)	15 (0%)	27 (100%)	–
Stage at diagnosis				**0.01**
IA or IB	7 (17%)	0 (0%)	7 (26%)	
IIA or IIB	23 (55%)	7 (47%)	16 (59%)	
IIIA or IIIB	11 (26%)	7 (47%)	4 (15%)	
Type of treatment				–
Curative	42 (100%)	15 (100%)	27 (100%)	
Age in years, median (IQR)	51 (44–59)	64 (54–67)	45 (42–53)	** < 0.001**
Owned cell phone	41 (98%)	14 (93%)	27 (100%)	0.36
Can receive and send text messages	40 (95%)	13 (87%)	27 (100%)	0.12
Preferred language				1
Setswana	35 (83%)	13 (87%)	22 (81%)	
English	7 (17%)	2 (13%)	5 (19%)	
Education level				0.06
None/non-formal	7 (17%)	4 (27%)	3 (11%)	
Primary	17 (40%)	7 (47%)	10 (37%)	
Junior secondary	10 (24%)	1 (7%)	9 (33%)	
Senior secondary	4 (10%)	0 (0%)	4 (15%)	
Tertiary	4 (10%)	3 (20%)	1 (4%)	
Occupation				0.12
Housewife	25 (60%)	12 (80%)	13 (48%)	
Employed (by someone)	15 (36%)	3 (20%)	12 (44%)	
Self employed	2 (5%)	0 (0%)	2 (7%)	
Marital status				**0.02**
Single	31 (74%)	8 (53%)	23 (85%)	
Married	8 (19%)	4 (27%)	4 (15%)	
Widowed	3 (7%)	3 (20%)	0 (0%)	
Number of children, mean (IQR)	3 (2–4)	3 (2–4)	3 (2–4)	0.88
Religion				1
None	4 (10%)	1 (7%)	3 (11%)	
Christian	38 (90%)	14 (93%)	24 (89%)	
Mode of transport to clinic				**0.04**
Own transport	3 (7%)	3 (20%)	0 (0%)	
Public transport	38 (90%)	12 (80%)	26 (96%)	
Other	1 (2%)	0 (0%)	1 (4%)	
*Screening history*				
Screened at least once prior to the last screen before diagnosis				1
No	20 (48%)	7 (47%)	13 (48%)	
Yes	10 (24%)	3 (20%)	7 (26%)	
Unknown	12 (29%)	5 (33%)	7 (26%)	
Symptoms on last screen before cancer diagnosis				1
Symptoms	25 (60%)	8 (53%)	17 (63%)	
No symptoms	6 (14%)	2 (13%)	4 (15%)	
Unknown	11 (26%)	5 (33%)	6 (22%)	
Days from last screen to diagnosis, median (IQR)	52.5 (15–176)	52.5 (15–176)	47 (9.5–177)	0.81
Days from last screen to diagnosis, mean (SD)	222 (442)	193 (337)	237 (493)	0.80

Bold values are statistically significant

Overall screening rates were low among participants regardless of their HIV status; only 24% reported to ever being screened prior to developing symptoms. At the screening visit most proximal to cancer diagnosis, 60% of participants had symptoms (Table 1). Across participants, the median time to diagnosis from their most proximal screening visit was 52 days (IQR 15–176), with no significant differences by HIV status.

General awareness and knowledge regarding cervical cancer screening was high in both groups, but knowledge about specific cervical cancer risk factors including HPV was low (Table 2). Only 62% of women knew HPV can cause cervical cancer and only 5% knew that HPV infection can be asymptomatic. In comparison to women living with HIV, women living without HIV were significantly less likely to know that women should be screened even if they are not experiencing symptoms (100% vs. 73%, *p* = 0.01). Regarding specific risk factors, women living with HIV tended to report higher levels of screening knowledge in comparison to women living without HIV overall (Mean (0–12): 8.9 vs 10.0, *p* = 0.05), with only one item reaching statistical significance: Women living with HIV were significantly more likely to know that vaginal washing (67% vs 27%; *p* = 0.01) did not increase cervical cancer risk than their counterparts. Table 2 summarizes all survey data from participants overall and by HIV status.

**Table 2:** Participant knowledge and beliefs

	Total	Characteristics by HIV status		
	All (n = 42)	Living without HIV (n = 15)	Living with HIV (n = 27)	*P* value
Cervical cancer prevention and screening awareness (% yes)				
Have you ever heard of cervical cancer?	41 (98%)	14 (93%)	27 (100%)	0.36
Have you ever heard of cervical cancer screening?	40 (95%)	14 (93%)	26 (96%)	1
Have you ever heard of a pap smear or a test where the doctor looks at a little piece of your cervix?	38 (90%)	11 (73%)	27 (100%)	**0.01**
Have you ever heard of visual inspection with acetic acid (VIA) or the vinegar test?	22 (52%)	6 (40%)	16 (59%)	0.34
Have you heard of human papillomavirus (HPV) vaccine?	15 (36%)	4 (27%)	11 (41%)	0.51
Do you know anyone who has been screened for cervical cancer?	17 (40%)	5 (33%)	12 (44%)	0.53
Cervical cancer screening and risk knowledge (% correct)				
Screening tests look for changes on your cervix that indicate you are at risk for cervical cancer (T)	39 (93%)	15 (100%)	24 (89%)	0.18
Women should get screened for cervical cancer only if they have symptoms (F)	38 (90%)	11 (73%)	27 (100%)	**0.01**
If a woman has abnormal vaginal bleeding…she should see a medical provider to get screened (T)	42 (100%)	15 (100%)	27 (100%)	
Cervical cancer can be prevented (T)	40 (95%)	14 (93%)	26 (96%)	0.67
Screening tests can help prevent cervical cancer (T)	40 (95%)	15 (100%)	25 (93%)	0.28
There is no treatment for cervical cancer (F)	29 (69%)	10 (67%)	19 (70%)	0.80
Family planning increases a woman’s risk of cervical cancer (F)	19 (45%)	4 (27%)	15 (56%)	0.07
Having HIV increases a woman’s risk of cervical cancer (T)	31 (74%)	9 (60%)	22 (81%)	0.13
Only HIV + women are at risk of getting cervical cancer (F)	33 (79%)	11 (73%)	22 (81%)	0.54
Women can lower their risk of cervical cancer by washing inside their vagina (F)	22 (52%)	4 (27%)	18 (67%)	**0.01**
Women can lower their risk of cervical cancer by getting a screening test (T)	41 (98%)	15 (100%)	26 (96%)	0.45
Women can do nothing to prevent cervical cancer because it is fate or God’s will (F)	31 (74%)	11 (73%)	20 (74%)	0.96
HPV-specific knowledge (% correct)				
HPV is an infection that can cause cervical cancer (T)	26 (62%)	10 (67%)	16 (59%)	0.64
HPV is spread by close contact between humans like during sexual intercourse (T)	24 (57%)	8 (53%)	16 (59%)	0.71
People with an HPV infection will always have vaginal symptoms (F)	2 (5%)	1 (7%)	1 (4%)	0.67
HIV awareness				
Have you heard about the disease known as HIV and AIDS? (Y)	42 (100%)	15 (100%)	27 (100%)	
Is HIV and AIDS a communicable disease? (Y)	41 (98%)	15 (100%)	26 (96%)	1
Is HIV the infection that causes AIDS? (Y)	41 (98%)	15 (100%)	26 (96%)	1
Does AIDS affect the immune system? (Y)	42 (100%)	15 (100%)	27 (100%)	–
HIV transmission knowledge (% correct)				
By sexual intercourse (Y)	42 (100%)	15 (100%)	27 (100%)	–
Through witchcraft or other supernatural means (N)	40 (95%)	14 (93%)	26 (96%)	0.67
From mother to child (Y)	41 (98%)	15 (100%)	26 (96%)	0.45
By sharing needle or syringe (Y)	40 (95%)	14 (93%)	26 (96%)	0.67
By blood transfusion (Y)	35 (83%)	11 (73%)	24 (89%)	0.19
By shaking hands (N)	36 (86%)	13 (87%)	23 (85%)	0.90
By eating from same plate or drinking from same glass as a person with HIV (N)	37 (88%)	13 (87%)	24 (89%)	0.83
By wearing the same clothes as a person with HIV (N)	38 (90%)	13 (87%)	25 (93%)	0.53
By a bite from a mosquito or other insect (N)	13 (31%)	3 (20%)	10 (37%)	0.25
Through contact with a doctor, dentist, or other health care professional (N)	32 (76%)	9 (60%)	23 (85%)	0.07
Through a curse (N)	30 (71%)	11 (73%)	19 (70%)	0.84
As a punishment from God (N)	35 (83%)	14 (93%)	21 (78%)	0.19
HIV prevention knowledge (% correct)				
By not sharing needles, syringes, or apparatus to inject drugs, vitamins, hormones, steroids, or medicine (Y)	39 (93%)	14 (93%)	25 (93%)	0.93
HIV can be prevented by using condoms properly during sexual intercourse (Y)	41 (98%)	15 (100%)	26 (96%)	0.45
HIV transmission can be avoided by remaining faithful to a single partner (Y)	42 (100%)	15 (100%)	27 (100%)	–
HIV transmission can be avoided by having a blood test before marriage (N)	0 (0%)	0 (0%)	0 (0%)	–
HIV can be prevented by avoiding blood transfusions (Y)	19 (45%)	8 (53%)	11 (41%)	0.43
HIV can be prevented by abstinence (no sex at all) (Y)	38 (90%)	15 (100%)	23 (85%)	0.12
HIV can be prevented by no casual sex (Y)	42 (100%)	15 (100%)	27 (100%)	–
HIV can be prevented by no commercial sex (Y)	42 (100%)	15 (100%)	27 (100%)	–
HIV can be prevented by having fewer partners (Y)	42 (100%)	15 (100%)	27 (100%)	–
Summed knowledge (higher = more items correct)				
Cervical cancer screening factors (0–12), mean (SD)	9.6 (1.8)	8.9 (1.9)	10.0 (1.5)	0.05
HIV transmission factors (0–12), mean (SD)	10.0 (1.5)	9.7 (1.5)	10.1 (1.5)	0.32
HIV prevention factors (0–9), mean (SD)	7.3 (0.8)	7.5 (0.5)	7.1 (0.9)	0.20

Bold values are statistically significant

### Exploring experiences and drivers of delay: appraisal, help-seeking, and diagnostic phases

Across the initial three phases of the Model of Pathways to Treatment (Appraisal, Help-Seeking, and Diagnostic Phases) [21], patients reported a variety of individual, community, and system-level factors that both hindered and enhanced access to timely care. Table 3 outlines thematic quotes from various phases in Model of Pathways to Treatment in addition to the sections below.

**Table 3:** Participant thematic quotes

Domain/themes	MPT phase	Exemplary quotes (in addition to those listed in the text)
Limited awareness of screening as prevention	Appraisal	*Higher awareness*: When you test [the] first time and you are negative it does not mean you it will never be there. You have to keep on going back to test for whether it is there or it is not (Early-50 s, PLWH) *Lower awareness*: According to what I heard; unprotected sex can cause cervical cancer. To prevent it you can consult with the doctors so they can identify whether you have the illness or not and give you treatment such as chemotherapy or radiation (Late-40 s, PLWH)
Limited knowledge about HPV	Appraisal	Yes ma’am. I am disappointed to say this. I do not understand about the [HPV] virus…I have not come to know of it (Early-50 s, PLWH) What I have heard is that cervical cancer can be caused by having sex at a younger age...Cervical cancer can be prevented by refraining from sex while still young...I have no idea what causes HPV and how it can be prevented (Early-50 s, PLWH) I would not want to lie to you and say I have heard anything [about what causes cervical cancer]. What I know is while listening to people with cervical cancer, us women, most people thought it was witchcraft (Late-60 s, PLWH)
High HIV-related awareness	Appraisal	With HIV I it is an issue I could say is like a national anthem we always hear about on the radio. Every time even on the radio we are taught about it. (Late 60 s, PLWH) HIV is transmitted through unprotected sexual intercourse, having multiple sexual partners, and getting into marriages without getting tested…It is desirable that when realize that children are starting to mature we teach them about HIV transmission and that it can be prevented through protected sex and going for testing. Now we can prevent it through being keen on testing and protecting ourselves. (Early-50 s, PLWH) I have heard that it is caused by having multiple sex partners, sharing needles with HIV positive individuals…It can be prevented by sticking to one partner and avoiding sharing needles. (Early-40 s, PLWH) Yes, ma’am it can be prevented by not having unprotected sexual intercourse. When you have sex you have to use condoms. (Early-40 s, PLWH)
Cancer fear and fatalism	Appraisal/help-seeking	I am not someone who visits hospitals regularly [and] hear people speaking ill of [hospital]. As if to say when you go to [the hospital] you are lost. (Late-60 s, PLWH)
	Help-seeking	[After hearing my diagnosis] my emotions became low, and I would ask myself what the way forward is. What is going to happen in future? Am I going to find that it has become worse? Or at what stage is it going to be in future? (Early-30 s, PLWH) After I was told the results, I became emotionally mixed up. I lost faith. I just saw death in me because we are used to knowing that when you hear that someone has cancer you know that they are going to die. This is what I thought of until I received treatment…that is when I had hope (Late 40 s, PLWOH)
Structural and individual barriers to seeking care	Help seeking	I do not know how to explain those reasons, but I kept on postponing. I couldn’t get leave days from my employer because she complained of my absence in the workplace. In one instance I lost my job because I had to go for check-ups and the employer could not keep up with it (Late-40 s, PLWH)
	Diagnostic	It is difficult [to get healthcare services] because I have to use public transport. I see that I just have to make a decision and appeal to my children… It is slightly difficult because I have to first tell my children that this is when [my appointment is] and then they would then rush to assist (Early-70 s, PLWOH)
Delays in receipt of results	Diagnostic	But I had tested because I was unwell and was not feeling well. My results were unavailable. I then remained for a while. I continued to feel that my health was deteriorating (Early-60 s, PLWOH) I had spent a long time waiting for results because I had spent years testing for this virus trying to find out what it is whether it is AIDS or it is the cancer… They tested me and the papers disappeared. The result had also disappeared. This and that… I was not receiving the results [of the screening tests]. (Late-60 s, PLWOH)
Social and religious sources of support	All phases	I was so scared thinking that was the end of my life…[but] I received very great support from my family and the health providers. (Early-50 s, PLWH) “I was sad but soon told myself, “Nowadays it is better it is no longer like the old days.” … “Nowadays the doctors are here.” My emotions then became better. I became faithful and thought of God. I thought of God that God is here. When they were treating me and put me where they put me, I will accept. But I was saying this to myself while also praying (Late-60 s, PLWOH)
System-level support	All phases	I do not experience any difficulty because if I want services, I seek them…. I just go and receive consultation…Because I do not hide. (Late-50 s, PLWOH) “It is very easy [to seek health services] … My health comes first, and it is my main concern” (Late-60 s, PLWOH)

MPT, model of pathways to treatment; PLWH, person living with HIV; PLWOH, person living without HIV

#### Appraisal: screening awareness and symptom assessment

Aligned with quantitative findings, many participants expressed limited awareness of risk factors that can contribute to cervical cancer, particularly HPV, throughout the interviews. This is exemplified in the response of one participant who stated “Honestly, I cannot talk about HPV because it is the first time I hear the name HPV, and this is what would make it difficult for me to answer.” (Early-50 s, Person Living with HIV [PLWH]).

Similarly, few participants knew that cervical cancer could be prevented through screening and thus often described screening as a test to find out if they have cancer or if they were experiencing symptoms, as reflected by these two participants:

“I have not heard of anything that causes cervical cancer. I do not know how it can be prevented but I heard people mentioning that for one to know if they have cervical cancer, they have to go for cancer screening.” (Early-40 s, PLWH).

“I once heard that when experiencing a lot of unpleasant vaginal discharge, one should check the doctors for cervical cancer screening.” (Late-50 s, PLWH).

One participant further explained that she was unaware of the potential severity of cervical cancer, noting: “Now I know the danger of cervical cancer. Back then I did not know. When people talked about cancer and its existence, I never paid attention. I did not know anything.” (Late 40 s, PLWH).

#### Help-seeking: undergoing screening and diagnostic evaluation

Many participants expressed individual fears or concerns as barriers to screening and cancer care including fear of going to the hospital.

“I would often, in my life like I was telling you that I am not someone who visits hospitals regularly, hear people speaking ill of [the local hospital]. As if to say when you go to [this hospital] you are lost.” (Late-60 s, Person Living Without HIV [PLWOH]).

While fear shaped delays for some, other participants generally noted avoidance of care until a pressing symptom arose as a driver of delays.

“Sometimes we tend to brush off some issues. We delay [screening] and only become alert when the signs start showing.” (Late-50 s, PLWH).

In addition to individual fears or avoidance of care, structural factors including limited testing equipment and other resources, added to challenges in screening and diagnosis as noted by this participant:

“I once went to the clinic and for us with HIV. We are supposed to have tested for cervical cancer before seeing a doctor. I went there and when it was my turn we were told that there was no testing equipment… The resources should always be available especially for patients living with HIV because we are too vulnerable to some illnesses.” (Early-40 s, PLWH).

#### Diagnosis: receiving results and follow-up care

Across the participants, the most substantial driver of delays seemed to be delays in both receipt of screening results and delays in appointments following abnormal screening. Many participants stated that they did not receive the results at all, even after multiple screenings. As one woman expressed when explaining why she eventually stopped going to get screened: “I once screened when I went for a six-week ante-natal checkup in [other city]. I always tested but did not receive any results until I gave up” (Early-40 s, PLWH).

Women also reported delays in returning for appointments following abnormal screening, even though they were recommended.

“The [screening] results were not good [two years before]. I am the one who delayed to go to the doctor… [until two years later when] I went back to the nurse to say, ‘I have not taken any measures since.’” (Early-50 s, PLWH).

#### Cross-cutting facilitators

Amid discussions of the various challenges that women faced, many women described sources of support and strength that helped them to overcome these challenges. Several women noted that they drew upon their community including their children and their religious community if they were in need. For example, one woman stated:

“I could not accept the results very well, it was so difficult, my children sat me down and explained that cancer is like any other disease, and I could live longer if I go for checkups regularly.” (Late-50 s, PLWH).

Other women noted that system-level or community services were available and accessible but required that they seek these services out.

“It is not difficult [to seek healthcare services] because some of us know the illnesses we have and where to go for assistance at the clinic. When you are given something to take for your ailment you also know what to do… It is easy because I have already taken a step towards it. I have not hidden my medical condition” (Early-50 s, PLWH).

## Discussion

This study provides insight on patient-reported factors shaping delays in timely screening and follow-up care among women with cervical cancer in Botswana. Only 24% of women reported ever being screened prior to presenting with symptomatic disease. While other clinic-based studies in Botswana have indicated higher rates of screening (as high as 72% ever screened in one study) [11], our results indicate that many women diagnosed with cervical cancer are not undergoing screening prior to cancer diagnosis, which is a missed opportunity to increase early detection and improve survival [9, 11]. Although Botswana implemented a national screening program in 2012 and there have been concerted efforts to integrate cervical cancer screening into HIV care, our results indicate that screening remains suboptimal even for women who are receiving HIV care [7]. Screening prevalence are generally lower in sub-Saharan Africa. Two population-based surveys have reported screening prevalence rates for sub-Saharan countries (16.9 and 19.0%), with even lower rates of 4.8% and 6% in some country-specific reports [23–27].

Our qualitative analysis showed that various challenges exist in the screening journey for women in Botswana. Amid the numerous barriers, some participants also noted different sources of both social and structural support that helped to mitigate or at least reduce the burden of care. Despite some sources of support, individual factors such as fear of cancer and treatment, limited awareness of treatment options, and system-level delays, such as delays in obtaining results, contribute to overall challenges. Fear of cancer as a barrier to screening in sub-Saharan Africa has been reported by several authors, specifically as most women consider cancer to have no treatment [28, 29]. These women also reported fear or embarrassment of undergoing the screening test itself, and abandonment after a cancer diagnosis. Judgmental attitudes by health care providers have also been reported as a contributing factor to this fear [30]. System-level delays are a common occurrence in sub-Saharan Africa which leads to delays in treatment initiation and acts as a deterrent for utilizing screening services [29, 30].

In comparison to studies in the general population in Africa, we observed high rates of cervical cancer knowledge, which has also been noted in other studies looking at awareness of cervical cancer screening in Botswana [11, 31]. Women included in our study have undergone treatment for cervical cancer already and therefore it is likely that they discussed screening and risk factors in greater detail with their providers than women who have not been diagnosed with cancer. However, despite being diagnosed with cervical cancer, knowledge regarding HPV as a risk factor was low. Poor knowledge of HPV has also been reported by other authors in the region [32]. During the interviews, women often described screening more as a diagnostic test to find out if one had cancer rather than part of preventive care. A targeted campaign focused on increasing awareness of HPV as the cause of cervical cancer, but not as a cancer diagnosis itself, may help to enhance knowledge and reduce potential fear of screening.

As indicated by other studies, women living with HIV had higher levels of screening knowledge [11, 33]. This difference might be partly stemming from women with HIV being more connected to preventive care as part of their routine HIV care. In contrast to higher income countries, like the US where delays are greater in those women living with HIV, being integrated into the HIV care infrastructure in Botswana may enable greater awareness and access to preventive care. However, as indicated by our results and the results of others, these differences in knowledge and awareness do not translate to differences in care delays as all women face substantial delays in diagnosis confirmation. These delays may be due in part to the lack of clinics where patients can receive diagnostic care across the country and centralized laboratory services [34, 35]. Furthermore, despite the enhanced focus on screening for women connected to HIV care, there is still a great need to increase screening in this higher risk population.

Given the myriad of factors driving delays in screening and follow-up care in Botswana, there is a great need to identify multifactorial approaches to reduce patient-level barriers while improving system-level processes to care coordination and communication. These efforts could include providing infrastructure for pathology labs to increase communication to referring clinics and to patients, or providing direct navigation for patients who have abnormal results. Additionally, the high levels of knowledge regarding HIV indicate that public campaigns about HIV are working well, and potentially could be an effective model to increase awareness about the importance of cervical cancer screening as prevention at an early age. The significant difference in age between women living with HIV and those without is substantial, and similar to other larger scale estimates. Our results indicate that while there is growing infrastructure for both HIV and cervical cancer care, more is needed to ensure women undergo regular cervical cancer screening and if diagnosed, receive their results and care in a timely manner.

### Limitations

In addition to its strengths, this study has limitations including potential recall bias given the timing of these interviews after diagnosis and selection bias given that we conducted the study in the context of a treatment clinic. Furthermore, while the use of in-depth interviews provides rich details on the experiences of women undergoing cervical cancer treatment that is often missing from larger studies, this method is not designed to evaluate generalizability of these experiences in the wider population and therefore this study cannot assess how experiences may differ across Botswana or other LMICs. While the sample size is robust and saturated for a mixed methods study, the sample size is limited in comparison to larger quantitative studies.

## Conclusions

Our results indicate that at each phase of the cervical cancer screening and diagnostic journey, patients faced a series of individual and structural barriers. For cancer screening to be most effective, there must be widespread awareness and knowledge that screening is needed as part of preventive care. Most women in our study, even those living with HIV, were symptomatic at the time of screening and described limited awareness that screening should be done when a woman is asymptomatic. This is in direct contrast to relatively high levels of HIV-related knowledge. Related, women reported limited awareness that HPV, the primary cause of cervical cancer, can be detected through screening before cancer has developed. Beyond awareness, individual fears added additional barriers to receiving screening and beginning treatment. Even for those women who had been screened, substantial delays in receipt of positive results remained, contributing further to diagnostic delays and uncertainties about the benefit of screening. Given the multitude of drivers of delays, there is great need for both individual and system-level approaches that increase awareness and timely receipt of screening and follow-up care, without which cervical cancer incidence and mortality will continue to be high in Botswana and other high-HIV prevalent LMICs.

## Data Availability

Restrictions apply to the availability of data used in this study due to consent processes and ethical challenges in truly de-identifying qualitative data. However, summary data may be available from corresponding author upon reasonable request and as allowable by regulatory processes.
